# The differential plasma and ruminal metabolic pathways and ruminal bacterial taxa associated with divergent residual body weight gain phenotype in crossbred beef steers

**DOI:** 10.1093/tas/txad054

**Published:** 2023-05-23

**Authors:** Modoluwamu Idowu, Godstime Taiwo, Taylor Sidney, Olanrewaju B Morenikeji, Andres Pech Cervantes, Zaira M Estrada-Reyes, Matthew Wilson, Ibukun M Ogunade

**Affiliations:** Division of Animal Science and Nutritional Science, West Virginia University, Morgantown, WV 26505, USA; Division of Animal Science and Nutritional Science, West Virginia University, Morgantown, WV 26505, USA; Division of Animal Science and Nutritional Science, West Virginia University, Morgantown, WV 26505, USA; Division of Biological and Health Sciences, University of Pittsburgh, Bradford, PA 16701, USA; Agricultural Research Station, Fort Valley State University, Fort Valley, GA, USA; North Carolina Agricultural and Technical State University, Greensboro, NC 27411, USA; Division of Animal Science and Nutritional Science, West Virginia University, Morgantown, WV 26505, USA; Division of Animal Science and Nutritional Science, West Virginia University, Morgantown, WV 26505, USA

**Keywords:** feed efficiency, metabolomics, rumen microbiome, steroid hormone

## Abstract

We applied ruminal and plasma metabolomics and ruminal 16S rRNA gene sequencing to determine the metabolic pathways and ruminal bacterial taxa associated with divergent residual body weight gain phenotype in crossbred beef steers. A group of 108 crossbred growing beef steers (average BW = 282.87 ± 30 kg) were fed a forage-based diet for a period of 56 d in a confinement dry lot equipped with GrowSafe intake nodes to determine their residual body weight gain (RADG) phenotype. After RADG identification, blood and rumen fluid samples were collected from beef steers with the highest RADG (most efficient; *n* = 16; 0.76 kg/d) and lowest RADG (least efficient; *n* = 16; −0.65 kg/d). Quantitative untargeted metabolome analysis of the plasma and rumen fluid samples were conducted using chemical isotope labelling/liquid chromatography–mass spectrometry. Differentially abundant metabolites in each of the plasma and rumen fluid samples between the two groups of beef steers were determined using a false discovery rate (FDR)-adjusted *P*-values ≤ 0.05 and area under the curve (AUC) > 0.80. Rumen and plasma metabolic pathways that were differentially enriched or depleted (*P* ≤ 0.05) in beef steers with positive RADG compared to those with negative RADG were determined by the quantitative pathway enrichment analysis. A total of 1,629 metabolites were detected and identified in the plasma of the beef steers; eight metabolites including alanyl-phenylalanine, 8-hydroxyguanosine, and slaframine were differentially abundant (FDR ≤ 0.05; AUC > 0.80) in beef steers with divergent RADG; five metabolic pathways including steroid hormone biosynthesis, thiamine metabolism, propanoate metabolism, pentose phosphate pathway, and butanoate metabolism were enriched (*P* ≤ 0.05) in beef steers with positive RADG, relative to negative RADG steers. A total of 1,908 metabolites were detected and identified in the rumen of the beef steers; results of the pathway enrichment analysis of all the metabolites revealed no metabolic pathways in the rumen were altered (*P* > 0.05). The rumen fluid samples were also analyzed using 16S rRNA gene sequencing to assess the bacterial community composition. We compared the rumen bacterial community composition at the genus level using a linear discriminant analysis effect size (LEfSe) to identify the differentially abundant taxa between the two groups of beef steers. The LEfSe results showed greater relative abundance of *Bacteroidetes*_vadinHA17 and *Anaerovibrio* in steers with positive RADG compared to the negative RADG group, while steers in the negative RADG group had greater relative abundance of *Candidatus_Amoebophilus*, *Clostridium_sensu_stricto_1*, *Pseudomonas*, *Empedobacter*, *Enterobacter*, and *Klebsiella* compared to the positive RADG group. Our results demonstrate that beef steers with positive or negative RADG exhibit differences in plasma metabolic profiles and some ruminal bacterial taxa which probably explain their divergent feed efficiency phenotypes.

## INTRODUCTION

Feed efficiency continues to be of great economic importance in animal production due to increasing feed costs (Koch et al., 1963; Herd et al., 2003). Phenotypic and genetic selection based on measures of feed efficiency is becoming a common practice to reduce the cost of animal production. Two of the most common measures of feed efficiency in beef cattle include residual feed intake (RFI) and residual body weight gain (RADG). Residual feed intake is the difference between an animal’s actual feed intake and its predicted feed intake on the basis of its production and maintenance requirements (Koch et al., 1963; Herd et al., 2004) and is calculated by regressing feed intake on body weight and body weight gain (Berry and Crowley, 2012). Using RFI, feed efficient animals are known to consume less feed than expected therefore having negative RFI values, while animals considered inefficient consume more feed than expected and have positive RFI values. Residual body weight gain is similar in principle to RFI except that ADG is regressed on feed intake and BW in the calculation of RADG (Crowley et al., 2010). Thus, RADG is associated with faster growth rates with no differences in DMI (Berry and Crowley, 2012). Greater or positive RADG values indicate efficient animals and are more desirable, while lesser or negative RADG values indicate inefficient animals (Crowley et al., 2010).

Due to the economic importance of feed efficiency, some recent studies have investigated the impact of divergent RFI phenotype on host metabolism, immunity, and rumen microbiome (Liu et al., 2022; Taiwo et al., 2022a). The results of these studies revealed that divergent RFI phenotype is associated with altered plasma and ruminal metabolites, ruminal microbiome, and hepatic and whole-blood genes related to protein metabolism and stress responsiveness (Liu et al., 2022; Taiwo et al., 2022b). However, to the best of our knowledge, the effects of divergent RADG phenotypes on host metabolism, rumen microbial taxa abundance and metabolome of beef cattle are currently not available. Therefore, we applied ruminal and plasma metabolomics to determine the metabolic pathways and 16S rRNA gene sequencing, a widely used approach in microbiology to study microbial communities and diversity, to determine the ruminal microbial taxa associated with divergent RADG phenotype in crossbred beef steers. We hypothesized that beef steers with divergent RADG phenotypes would exhibit differences in host metabolome and rumen metabolome and microbial community.

## MATERIALS AND METHODS

### Animals, Feeding, RADG Determination, and Sample Collection

The research procedures were approved by the Institutional Animal Care and Use Committees of West Virginia (protocol number 2204052569). A group of 108 crossbred growing beef steers (average BW = 282.87 ± 30 kg) were fed a high-forage total mixed ration (TMR; primarily consisting of corn silage; ground hay; and a ration balancing supplement; CP = 13.2%, NDF = 45.9%, and NEg = 0.93 Mcal/kg; calculated based on NASEM (2016) using measured values of animal BW, dry matter intake, and diet information) in five dry lot pens (20–22 steers per pen) each equipped with two GrowSafe 8000 intake nodes (GrowSafe Systems Ltd., Airdrie, Alberta, Canada) for a period of 49 d to determine their RADG phenotype. Details of animal feeding and procedures have been reported in our previous study (Taiwo et al., 2022b). Briefly, the steers were allowed to acclimate to the feeding facilities and diet for a period of 15 days. Following this period, individual feed intake was measured over a span of 49 days. Daily BW for each animal, measured using In-Pen Weighing Positions (IPW, Vytelle LLC), was regressed on time using simple linear regression to calculate initial BW, mid-test BW, and average daily gain (ADG). The initial BW, mid-test metabolic BW, and ADG were calculated by regressing the daily BW for each animal using simple linear regression. The ADG of each steer was regressed against their daily dry matter intake (DMI) and mid-test metabolic BW (MMTW = mid-test BW^0.75^), and the RADG was calculated as the residual or the difference between the predicted value of the regression and the actual measured value based on the following equation: *Y* = *β*_0_ + *β*_1_*X*_1_ + *β*_2_*X*_2_ + *ε*, where *Y* is the ADG (kg/d), *β*_0_ is the regression intercept, *β*_1_ and *β*_2_ are the partial regression coefficients, *X*_1_ is the MMTW (kg), *X*_2_ is the observed DMI (kg/d) (Koch et al., 1963; Crowley et al., 2010).

At the end of the feeding trial, the steers were ranked based on their RADG coefficients. The most efficient steers with the greatest positive RADG (*n =* 16) and the least efficient steers with the least negative RADG (*n =* 16) were identified. Blood samples were collected weekly for three weeks from the coccygeal vessel of both groups of steers into 10-mL vacutainer tubes containing sodium heparin (Vacutainer, Becton Dickinson, Franklin Lakes, NJ). Immediately after collection, the blood samples were placed on ice and were centrifuged at 2,500 × *g* for 15 min at 4 °C to harvest the plasma. The plasma samples were then frozen at −20 °C, later thawed at room temperature and then composited for each steer. The composite samples were then stored at −80 °C until further analysis.

Rumen fluid samples (500 mL) were also collected from each steer weekly for three weeks at about 4 hr after feeding using an orally administered stomach tube connected to a vacuum pump. The stomach tube is 260 cm in length and 1.90 cm in diameter. The tube was connected to a probe head (12 cm long, 3 cm in diameter, 160 holes) at one end and to a pumping system at the other. The steers were restrained in the chute, and the probe end of the stomach tube was inserted to a depth of 180 cm, which is an appropriate length to reach the rumen content. About 500 mL of rumen fluid was extracted using the pump after discarding the first 200 mL of rumen fluid to reduce saliva contamination. A subsample of the rumen content was manually homogenized and stored immediately at −80 °C until DNA extraction and sequencing were performed.

### DNA Extraction and 16S rRNA Gene Sequencing

After thawing at room temperature, the rumen fluid samples were composited for each beef steer. Microbial DNA was extracted from the rumen fluid samples (0.25 g) after thawing at room temperature using a Qiagen DNeasy Powersoil DNA Isolation kit following the manufacturer’s instructions (Qiagen; catalog number: 47014). Total DNA concentration was measured using a NanoDrop 2000 spectrophotometer (Thermo Fisher Scientific, Waltham, MA) with an A260:A280 ratio from 1.8 to 2.0 (Thermo Fisher Scientific, Waltham, MA). The samples were prepared using Qiagen QIAseq phased primers that targeted the V3/V4 regions of the 16S gene following the manufacturer’s instruction (Qiagen; catalog number: 333845). The forward primer sequence was 5ʹ-CCTACGGGNGGCWGCAG-3ʹ and the reverse primer sequence was 5ʹ-GACTACHVGGGTATCTAATCC-3ʹ. After cleaning and normalization, the samples were sequenced on a v3 MiSeq 600-cycle flowcell to generate 2 × 276 bp PE reads.

### Metabolome Analysis of Rumen Fluid and Plasma Samples

Quantitative untargeted metabolome analysis of the rumen fluid and plasma samples was performed using a chemical isotope labelling (CIL)/liquid chromatography–mass spectrometry (LC–MS)-based technique (Zhao et al., 2019). This technique uses ^12^C and ^13^C-isotope dansylation labelling to detect metabolites based on their chemical groups such as amine/phenols (metabolites associated with amino acid metabolism), carboxylic acid (metabolites associated with metabolism of fatty acids and their derivatives), carbonyl (majorly aldehydes and ketones), and hydroxyls (Zhao et al., 2019). Detailed information of the technique including sample preparation and analysis has been published in previous studies (Mung and Li, 2017; Zhao and Li, 2021). Briefly, each sample was vortexed and then centrifuged at 15,000 × *g* for 5 min. A 100 µL pre-aliquot was taken from the supernatant of each sample to reduce the number of freeze–thaw cycles. The total metabolite concentration of each sample was determined by NovaMT Sample Normalization Kit (Nova Medical Testing Inc. Edmonton, Alberta, Canada; Wu and Li, 2016). Based on the quantification results, 100 µL pre-aliquots were then diluted to give each sample a concentration of 1 mM. Supernatants were taken from diluted pre-aliquots for different chemical isotope labelling and preparation of pooled samples (a mixture of 50 µL of aliquots taken from each sample). After protein precipitation (addition of 90 μL of LC–MS grade methanol, and then incubation at −20 °C for 0.5 h), chemical isotope labelling (^12^C and ^13^C-isotope dansylation labeling described by Zhao et al., 2019), and preparation of quality control samples (equal volume mixture of a ^12^C-labeled and a ^13^C-labeled pooled samples), all the samples were analyzed using LC linked to quadrupole time-of-flight MS (Agilent, Billercia, MA; Mung and Li, 2017). Chromatographic separations were performed on an Agilent eclipse plus reversed-phase C18 column (150 × 2.1 mm, 1.8 µm particle size) at an oven temperature of 40 ^o^C. Mobile phase A consisted of 0.1% (v/v) formic acid in water. Mobile phase B was 0.1% (v/v) formic acid in acetonitrile. The flow rate was 400 µL/min.

### Data and Statistical Analysis

#### Growth performance data.

Variables such as initial and final body weights, ADG, DM intake, and RADG values of the beef steers with positive RADG (*n* = 20) and negative RADG (*n* = 20) were analyzed using the GLIMMIX procedure of SAS version 9.4 (SAS Institute Inc., Cary, NC), with RADG status included as a fixed effect. Significant effects were declared at *P* ≤ 0.05. Values of initial body weight were included as a covariate for the final body weight.

#### 16S rRNA sequence data analysis.

Quality control and adapter trimming of the raw sequence files were performed using Illumina binary base call (BCL) Convert v3.9.3 (Illumina, San Diego, CA, USA) using default parameters. The fastq files were then imported into Qiime2 (Bolyen et al., 2019) for subsequent analysis. Primer sequences were removed using Qiime2’s cutadapt plugin (Martin, 2011). Sequences were denoised using Qiime2’s dada2 plugin (Callahan et al., 2016). Denoised sequences were assigned operational taxonomic units (OTUs) using the Silva database with a sequence similarity threshold of 97% and the VSEARCH () utility within Qiime’s feature-classifier plugin. The OTUs were collapsed into their taxonomic units, and their counts were converted to reflect their relative frequency within a sample. Statistical analyses of the OTU data were performed using the MicrobiomeAnalyst platform (microbiomeanalyst.ca; Chong et al., 2020). First, the data were rarefied to the minimum library size and normalized with cumulative-sum scaling (Vikram et al., 2017). Thereafter, the rarefied data were used to analyze alpha diversity (Chao1 index) and beta diversity (Bray-Curtis distance matrix-based principal coordinates analysis (PCoA) at the genus taxonomy level. The difference in beta diversity distance was also tested using Permutational Multivariate Analysis of Variance (PERMANOVA) at 999 permutations. Differential microbial taxa at the genus taxonomy level associated with divergent RADG phenotypes were identified using the linear discriminant analysis (LDA) effect size method (LEfSe) based on Kruskal–Wallis test of *α* ≤ 0.05 and logarithmic LDA score cut-off of 2.0.

#### Plasma and rumen metabolome analysis.

The MS spectral peaks from all samples (40 rumen fluid, 40 plasma, and 3 quality control samples) generated from the LC–MS were first exported with Agilent MassHunter software. The exported data were uploaded to IsoMS Pro 1.2.16 for data quality checks and processing (peak picking, peak pairing, and peak-pair filtering) to remove redundant peaks of the same metabolite such as adduct ions, dimers, and multimers. Peak pairs without data present in at least 80% of samples in any group were filtered out. After filtering, all data were normalized by the ratio of total useful signals. Metabolite identification of the peak pairs was done based on mass and retention time matching using CIL library (www.MyCompoundID.org) and based on accurate mass and predicted retention time matches using linked identity library which includes over 9,000 pathway-related metabolites (Li et al., 2013). The final metabolite-intensity data file was then exported to Metaboanalyst 5.0 software (Pang et al.,2021; https://www.metaboanalyst.ca/) for statistical analysis. Differentially abundant metabolites in each of the plasma and rumen fluid samples between the two groups of beef steers were determined using a false discovery rate (FDR)-adjusted *P*-values ≤ 0.05 and area under the curve (AUC) > 0.80 using a receiver operating characteristic (ROC) curve as calculated by the ROCCET web server after the data were log-transformed, auto-scaled, and normalized using median-scale normalization. Pathway enrichment analysis of the rumen and plasma metabolome data were performed to determine the rumen and plasma metabolic pathways that were differentially enriched or depleted (*P* ≤ 0.05) in beef steers with positive RADG compared to those with negative RADG.

## RESULTS

### Growth Performance

The results showing the growth performance of beef steers with divergent RADG phenotypes are shown in Table 1. The average RADG values of positive RADG and negative RADG steers were 0.76 and −0.65 kg/d, respectively. The initial BW and DMI were similar (*P* > 0.05) for the two groups; however, final body weight and ADG were greater (*P* = 0.01) in beef steers with positive RADG (1.25 kg/d) than those with negative RADG (0.94 kg/d).

**Table 1.** Growth performance of beef steers with divergent residual body weight gain phenotype

Item	Positive RADG	Negative RADG	SEM	*P*-value
RADG, kg/d	0.76	-0.65	0.10	0.01
Initial weight, kg	273	286	11.9	0.31
Final weight, kg	345	338	1.83	0.01
ADG, kg/d	1.29	0.94	0.09	0.01
DMI, kg/d	7.39	7.94	0.49	0.27

SEM, standard error of the mean; ADG, average daily gain; DMI, dry matter intake.

^a,b^Within a row, treatment means with different superscripts differ, *P* ≤ 0.05.

### Ruminal Bacterial Community

**Figure 1.**
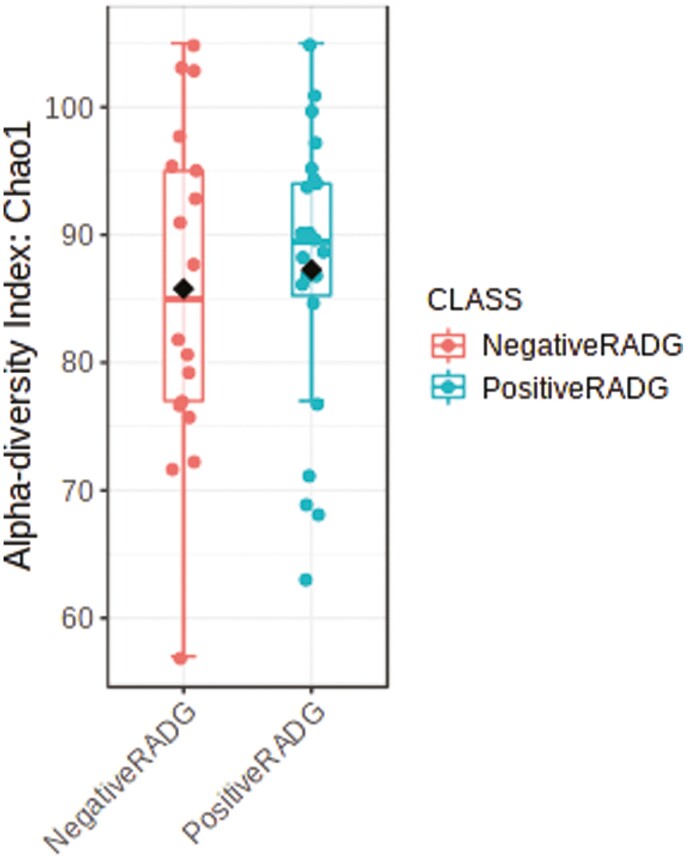
Alpha diversity (Chao1 index; *P*-value = 0.86) of the rumen bacterial community in beef steers with divergent residual body weight gain (RADG) phenotype. .

**Figure 2.**
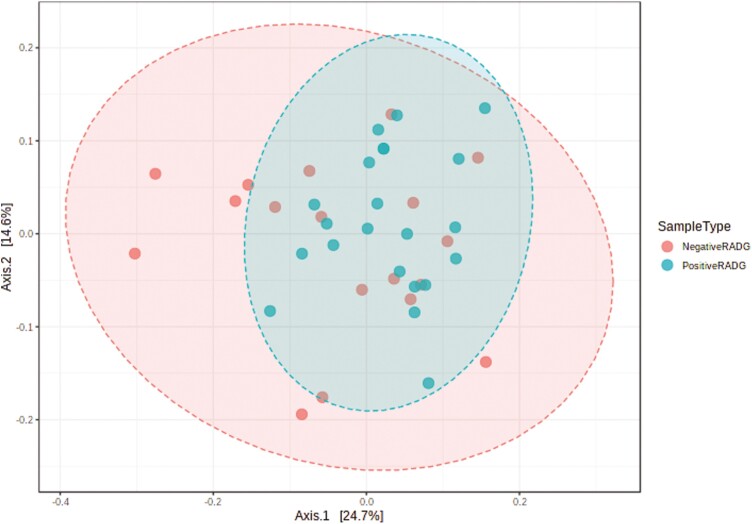
Beta diversity (Bray-Curtis-PCoA based on unweighted Unifrac distance) of the rumen bacterial community in beef steers with divergent residual body weight gain (RADG) phenotype (PERMANOVA *P*-value = 0.22).

**Figure 3.**
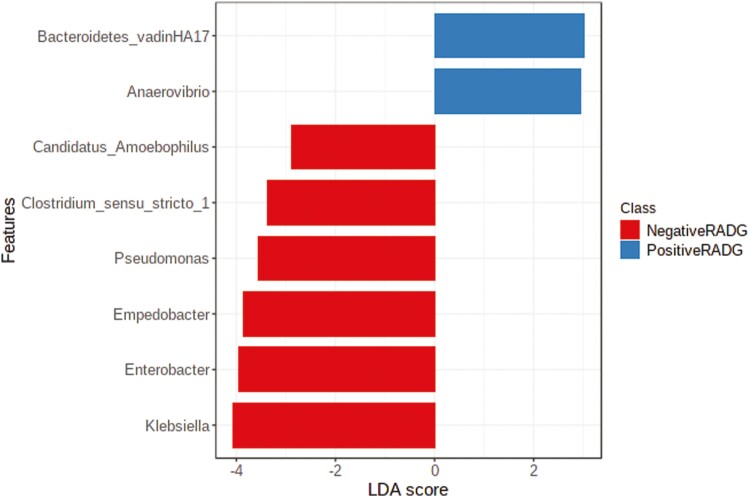
Differentially abundant bacterial taxa at the genus level determined using linear discriminant analysis effect size (LEfSe) analysis in beef steers with divergent residual body weight gain (RADG) phenotype.

There was an average of 86,681 ± 10,844 read pairs per sample. The sequence datasets analyzed in this study are all available in NCBI (BioProject number PRJNA955175). The rarefaction curves showed that the rate of increase in OTU number slowed down with the increasing reads per sample and tended to plateau, illustrating that the sequencing coverage was adequate (Supplementary Figure S1). The Chao1 index (a measure of alpha diversity) was similar (*P* = 0.86) for the two groups of beef steers (Figure 1). In addition, we observed no difference (*P* = 0.22) in beta diversity using the PLS-DA score plot based on an unweighted Unifrac distance (Figure 2). The LefSe results showed that the relative abundance of *Bacteroidetes*_vadinHA17 and *Anaerovibrio* were greater (LDA ≥ 2.0; *P* ≤ 0.05) in beef steers with positive RADG compared to the negative RADG group. Whereas the relative abundance of *Candidatus_Amoebophilus*, *Clostridium_sensu_stricto_1*, *Pseudomonas*, *Empedobacter*, *Enterobacter*, and *Kiebsiella* were lower (LDA ≥ 2.0; *P* ≤ 0.05; Figure 3).

### Ruminal Metabolome

**Figure 4.**
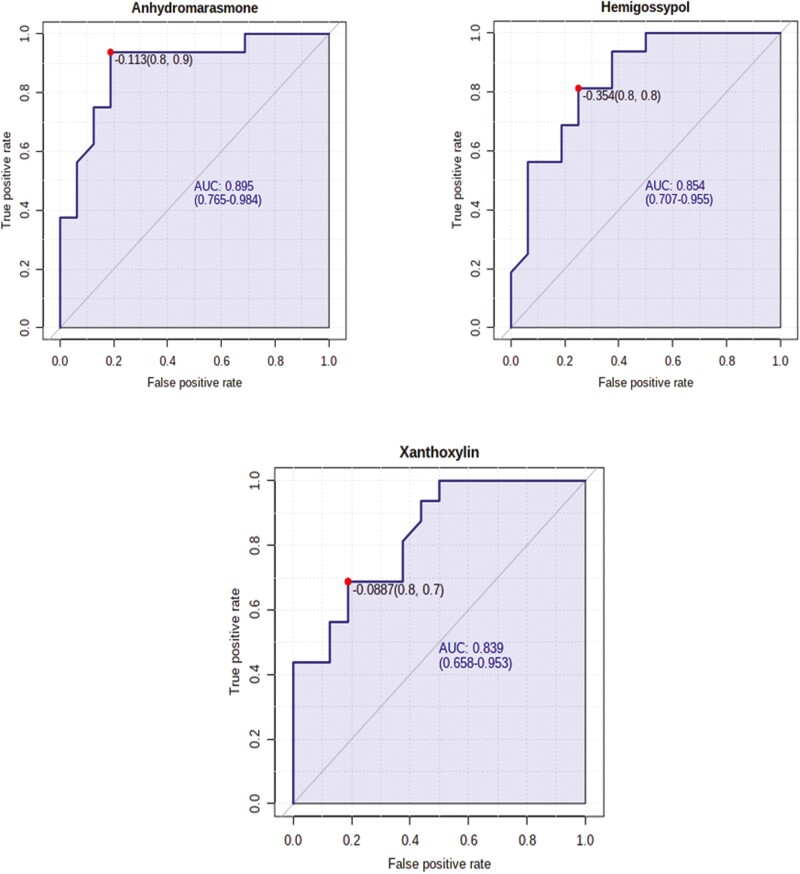
Biomarker analysis of the differentially abundant rumen metabolites in beef steers with with divergent residual body weight gain (RADG) phenotype. Only metabolites with AUC values > 0.80 are shown.

**Figure 5.**
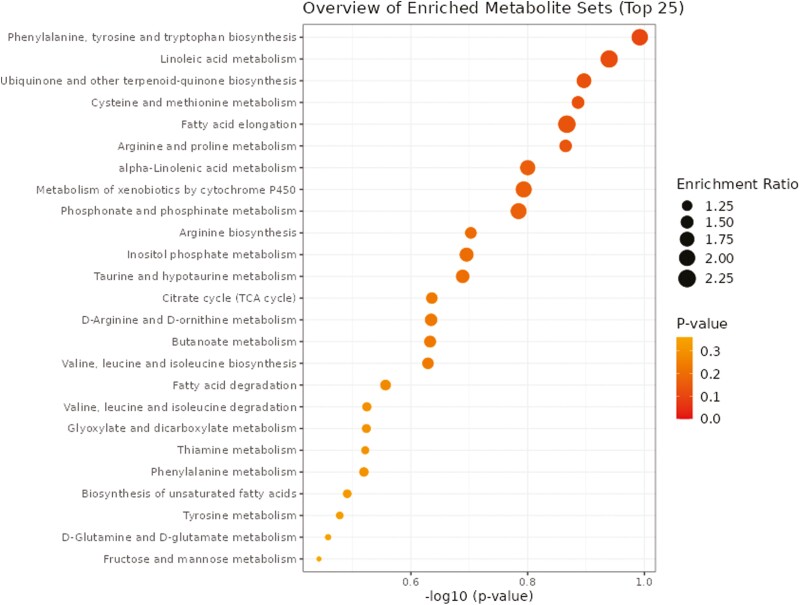
Pathway enrichment analysis of the rumen fluid metabolome. No metabolic pathways were altered (–log10(*P*) ≤ 1.3 (equivalent to *P* ≥ 0.05).

A total of 1,908 metabolites were detected and identified (Supplementary Table S1). The results of the ROC analysis revealed two metabolites (anhydromarasmone, hemigossypol) with respective AUC values of 0.89 and 0.85 were greater (FDR ≤ 0.05) in beef steers with positive RADG while one metabolite (xanthoxylin) with AUC value of 0.83 was greater (FDR ≤ 0.05) in beef steers with negative RADG (Figure 4). The box plots showing the distributions of these metabolites are shown in Supplementary Figure S2. Results of the pathway enrichment analysis of all the metabolites revealed no metabolic pathways were altered by divergent RADG phenotypes (*P* > 0.05) (Figure 5).

### Plasma Metabolome

**Figure 6.**
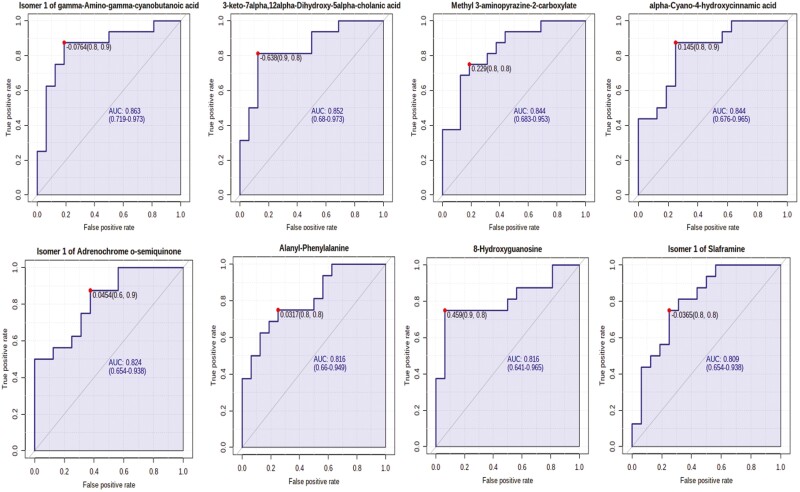
Biomarker analysis of the differentially abundant plasma metabolites in beef steers with divergent residual body weight gain phenotype. Only metabolites with AUC values > 0.80 are shown.

**Figure 7.**
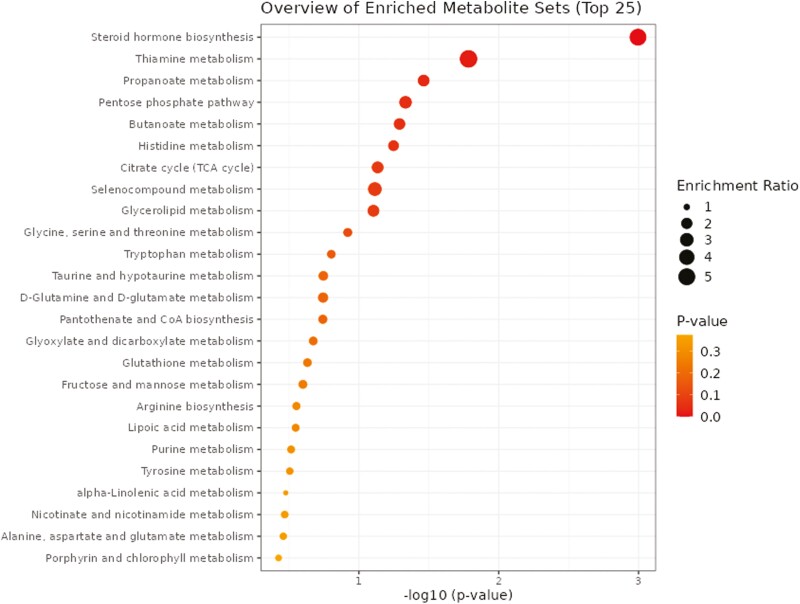
Pathway enrichment analysis of the plasma metabolome. Metabolic pathways with –log10(*P*) ≥ 1.3 (equivalent to *P* ≤ 0.05) are enriched in positive RADG steers, relative to negative RADG steers.

A total of 1,629 metabolites were detected and identified (Supplementary Table S2). The results of the ROC analysis revealed that five metabolites (cyanobutanoic acid, 3-aminopyrazine-2-carboxylate, hydroxycinnamic acid, alanyl-phenylalanine, and 8-hydroxyguanosine) with AUC values > 0.80 were greater (FDR ≤ 0.05) in beef steers with positive RADG while three metabolites (slaframine, adrenochrome o-semiquinone, and dihydroxy-cholanic acid) AUC values > 0.80 were greater (FDR ≤ 0.05) in beef steers with negative RADG (Figure 6). The box plots showing the distributions of these metabolites in the two groups of beef steers are shown in Supplementary Figure S3. Results of the pathway enrichment analysis of all the metabolites showed that five (5) pathways; steroid hormone biosynthesis, thiamine metabolism, propanoate metabolism, pentose phosphate pathway, and butanoate metabolism were enriched (*P* ≤ 0.05) in beef steers with positive RADG (Table 2; Figure 7).

**Table 2.** Enriched metabolic pathways and their associated plasma metabolites in beef steers with positive residual body weight gain phenotype

Metabolic pathways^1^	*P*-value	Associated metabolites
Steroid hormone synthesis	0.01	Dehydroepiandrosterone, cortisol, estrone
Thiamine metabolism	0.01	L-cysteine
Propanoate metabolism	0.03	Succinic acid, alanine, malonic semialdehyde, 2-hydroxybutyric acid, 2-ketobutyric acid, propionic acid, and hydroxypropionic acid
Pentose phosphate pathway	0.05	Glyceric acid; xylulose 5-phosphate; D-erythrose 4-phosphate
Butanoate metabolism	0.05	Gamma-aminobutyric acid, succinic acid semialdehyde; butyric acid, oxoglutaric acid; succinic acid

^1^Only metabolic pathways with *P* ≤ 0.05 are shown.

## DISCUSSION

The rumen microbiota comprises a vast range of microorganisms and metabolic products which are known to influence the performance, health, and feed efficiency of ruminants (McCann et al., 2014; Rodríguez, 2014; Miura et al., 2021). In this study, the relative abundance of *Bacteroidetes*_vadinHA17 and *Anaerovibrio* were greater in beef steers with positive RADG compared with those with negative RADG. *Bacteroidetes*_vadinHA17 belongs to the *Bacteroidetes* phylum. *Bacteroidetes* are known to express numerous genes that encode carbohydrate-active enzymes and therefore promote the degradation of complex carbohydrates through a series of metabolic pathways, thereby contributing to increased ruminal fiber degradation and energy release via propionate production (Flint and Duncan, 2014; Kirby et al., 2019; Larsbrink and McKee, 2020). Certain species of *Anaerovibrio* such as *Anaerovibrio lipolytica* are known to produce lipases for the hydrolysis of lipids to free fatty acids and glycerol, the latter of which is utilized by a wide variety of rumen micro-organisms to produce propionate (Hobson and Mann, 1961; Murphy, 2011; Mannelli et al., 2018). The greater relative abundance of *Bacteroidetes*_vadinHA17 and *Anaerovibrio* which are both associated with increased complex carbohydrate and lipid metabolism and increased energy release via propionate synthesis in the rumen suggest that beef steers with positive RADG have increased availability of nutrients and energy to support improved growth performance which explains their better feed efficiency compared with beef steers with negative RADG.

Results from this study showed that beef steers with positive RADG had lower relative abundance of *Candidatus Aemobophilus, Klebsiella*, *Pseudomonas*, and *Clostridum sensu stricto*. *Candidatus Aemobophilus* is an obligate intracellular symbiont of amoebas (Schmitz-Esser et al., 2008, 2010). Amoebas are known to be vectors and reservoirs of several disease agents (Albert-Weissenberger et al., 2007; Thomas and McDonnell, 2007). Zinicola et al. (2015) reported an increased relative abundance of *Candidatus Amoebophilus asiaticus* in cows with bovine digital dermatitis lesions, an infectious disease associated with lameness in cattle. Species of *Klebsiella* are gram-negative, rod-shaped bacteria that possess high degrees of virulence and can cause infections and antibiotic resistance in animals and humans (McCuddin et al., 2006). In dairy cows, *Klebsiella* spp. are one of the major causes of mastitis and reduced milk production (Zadoks et al., 2011). Certain species of *Enterobacter* such as *Enterobacter cloacae* are known to cause gastrointestinal infections, hence contributing to disease susceptibility in animals (Davin-Regli and PagÃ¨s, 2015). Furthermore, these bacterial species are able to acquire numerous genetic mobile elements that strongly contribute to antibiotic resistance (Davin-Regli and PagÃ¨s, 2015; Harada et al., 2017). *Pseudomonas* spp. are commonly known to be gram-negative, anaerobic, rod-shaped bacteria that can predispose ruminants to diseases due to their extreme antibiotic-resistant trait (Mulcahy et al., 2013; Diggle and Whiteley, 2020). *Clostridium* species are rod-shaped, gram-positive, anaerobic bacteria commonly found in the gastrointestinal tract of ruminants (Dykes, 2021). Species of *Clostridium* are divided into several clusters, including *Clostridium* cluster I, which represents the genus *Clostridium sensu stricto* (Collins et al. 1994). Within this cluster I, there are *Clostridium tetani* causing tetanus disease, *Clostridium chauvoei* which causes blackleg, a highly mortal disease of ruminants, and *Clostridium botulinum*, the producer of the strong botulinum neurotoxin leading to botulism in humans and animals (Lindström et al. 2010; Peck et al. 2011). The reduced relative abundance of the aforementioned rumen bacteria that can negatively impact the immune competence and performance of the beef steers by predisposing them to several diseases is evidence of a healthy rumen microbiome and health of beef steers with positive RADG; therefore, more energy will be available to support improved growth, which probably explains their better feed efficiency.

Metabolite pathway enrichment analysis of the ruminal and plasma metabolites was performed to better understand the metabolic pathways associated with divergent RADG phenotypes in the beef steers. Our results revealed that no ruminal metabolic pathway was altered. It is important to acknowledge that the identified differences in the abundance of certain genera, such as *Bacteroidetes_vadinHA17* and *Anaerovibrio*, were not explained by the rumen fluid metabolome data. There are several reasons why changes in rumen taxa may not always cause changes in the rumen metabolome. First, microbial metabolism in the rumen is highly complex, with many different pathways and interactions occurring simultaneously. Changes in the abundance of a single microbial taxon may not have a significant impact on overall metabolic activity if other microbial taxa are able to compensate for any metabolic changes. In addition, metabolomics analysis is limited by the sensitivity and resolution of the analytical methods used. It is possible that changes in the rumen metabolism may be occurring but are not detectable using the metabolomics analysis employed in this study.

Five metabolic pathways in plasma; steroid hormone biosynthesis, thiamine metabolism, propanoate metabolism, pentose phosphate pathway, and butanoate metabolism were enriched in beef steers with positive RADG, relative to those with negative RADG. Steroid hormones are synthesized from cholesterol and are secreted from either the adrenal cortex, testes, or ovaries (Heffner and Schust, 2010). The adrenal cortex produces glucocorticoids that play a huge role in glucose metabolism by amplifying the expression of enzymes such as glucose-6-phosphatase that trigger gluconeogenesis in the liver (Sapolsky, 1994). Glucocorticoids also function in stress response by possessing anti-inflammatory properties and several immune functions (Funder et al., 1996; Frye, 2009). Thiamine is essential to the health of all living organisms due to its fundamental role in energy metabolism (Edwards, 2022; NCBI, 2022). Thiamine serves as a cofactor for several enzymes including transketolase, α-ketoglutarate dehydrogenase, pyruvate dehydrogenase, and branched chain α-keto acid dehydrogenase (Lonsdale, 2006; Nabokina and Said, 2012; Mkrtchyan et al., 2015). These enzymes are involved in pathways such as the Krebs cycle that allows for the production of ATP, NADPH, and ribose-5-phosphate which are critical for generating cellular energy and downstream production of amino acids, nucleic acids, and fatty acids (Lonsdale, 2006; Sriram et al., 2012). Propanoate metabolism is essentially the metabolism of propionic acid which is produced in the form of its coenzyme A ester, propionyl-coA. Propionic acid functions as a substrate for hepatic gluconeogenesis through its conversion to succinyl-CoA (Aschenbach et al., 2010; Perry et al., 2016). The pentose phosphate pathway is an alternative energy-generating pathway that links carbohydrate and fatty acid metabolism, nucleotide synthesis and helps prevent oxidative stress (Alfarouk et al., 2020). Butanoate metabolism pathway generates enzymes responsible for acetyl-CoA biosynthesis which is required for ATP production in the mitochondria (Bailey et al., 2014). Taken together, the enrichment of these aforementioned pathways and their associated metabolites in beef steers with positive RADG suggests an increased efficiency of energy metabolism and synthesis availability and efficiency, thus allowing for greater body weight gain despite similar DMI relative to beef steers with negative RADG.

## CONCLUSION

Our results show some early evidence that suggest that beef steers with divergent RADG phenotypes exhibit differences in the relative abundance of some ruminal bacterial taxa and plasma metabolic profiles. Notably, the relative abundance of *Bacteroidetes*_*vadinHA17* and *Anaerovibrio* which are both involved in carbohydrate and lipid metabolism and propionate production were greater in beef steers with positive RADG. Similarly, energy and health-promoting pathways such as steroid hormone biosynthesis, thiamine metabolism, propanoate metabolism, pentose phosphate pathway, and butanoate metabolism were also enriched in beef steers with positive RADG compared to negative RADG.

## Supplementary Material

txad054_suppl_Supplementary_Figure_S1

txad054_suppl_Supplementary_Figure_S2

txad054_suppl_Supplementary_Figure_S3

txad054_suppl_Supplementary_Table_S1

txad054_suppl_Supplementary_Table_S2
