# Supplementary figures and images for: The differential plasma and ruminal metabolic pathways and ruminal bacterial taxa associated with divergent residual body weight gain phenotype in crossbred beef steers
Source: Transl Anim Sci. 2023 May 23;7(1):txad054. doi: 10.1093/tas/txad054 (PMC10332501; doi:10.1093/tas/txad054)

**
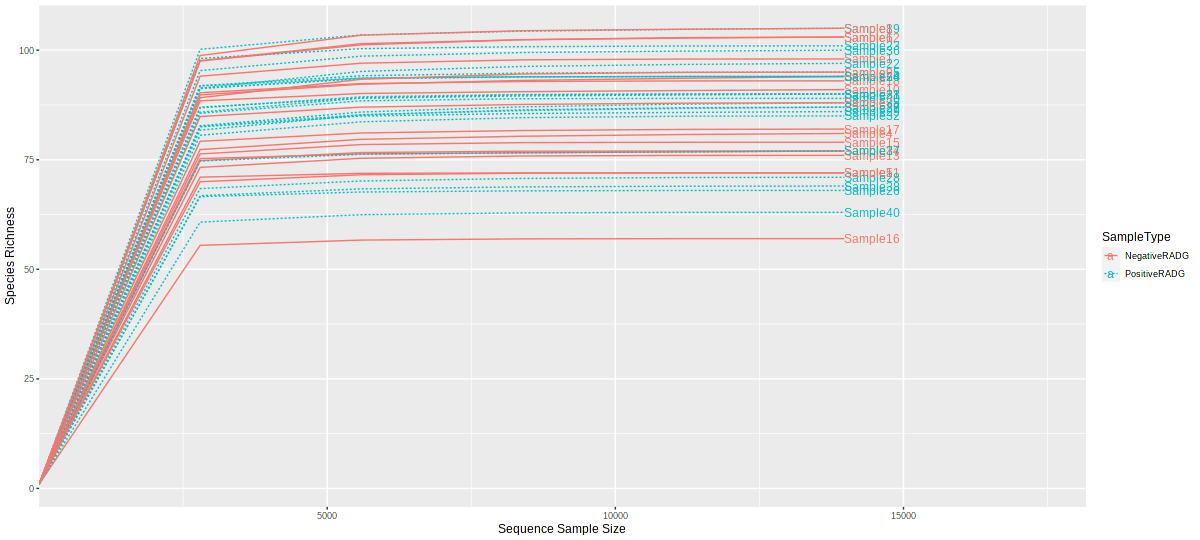
**

**Figure S1: Rarefaction curves of all the samples**

Supplement: txad054_suppl_Supplementary_Figure_S1 [file txad054_suppl_supplementary_figure_s1.docx]
