# Supplementary material for: The differential plasma and ruminal metabolic pathways and ruminal bacterial taxa associated with divergent residual body weight gain phenotype in crossbred beef steers
Source: Transl Anim Sci. 2023 May 23;7(1):txad054. doi: 10.1093/tas/txad054 (PMC10332501; doi:10.1093/tas/txad054)
Supplement: txad054_suppl_Supplementary_Figure_S2 [file txad054_suppl_supplementary_figure_s2.docx]

Anhydromarasmone

Hemigossypol

Xanthoxylin


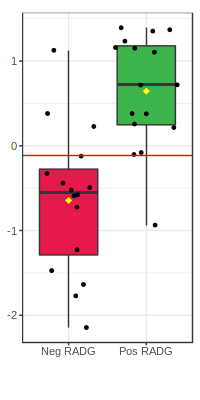

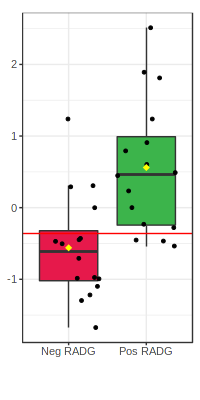

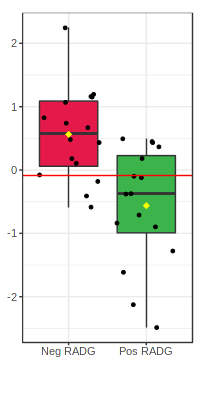


**Figure S2.** Relative distributions of the differentially abundant rumen metabolites in beef steers with divergent residual gain phenotype.
