# Supplementary material for: The differential plasma and ruminal metabolic pathways and ruminal bacterial taxa associated with divergent residual body weight gain phenotype in crossbred beef steers
Source: Transl Anim Sci. 2023 May 23;7(1):txad054. doi: 10.1093/tas/txad054 (PMC10332501; doi:10.1093/tas/txad054)
Supplement: txad054_suppl_Supplementary_Figure_S3 [file txad054_suppl_supplementary_figure_s3.docx]

Dihydroxy-cholanic acid

Hydroxycinnamic acid

3-aminopyrazine-2-carboxylate

Gamma-Amino-gamma-cyanobutanoic acid


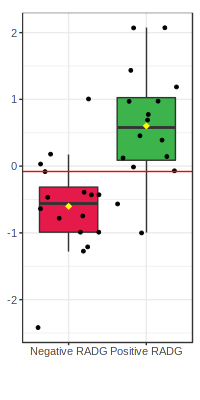

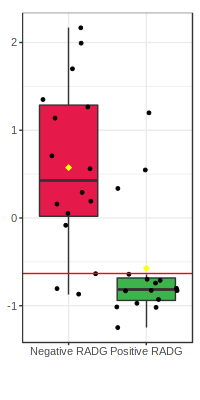

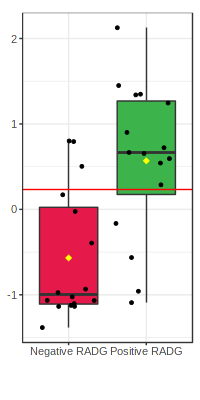

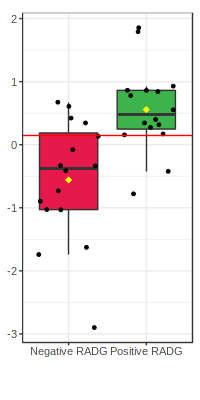


Adrenochrome o-semiquinone

Slaframine

8-Hydroxyguanosine

Alanyl-Phenylalanine


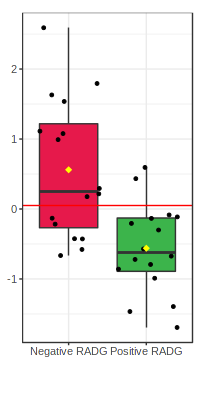

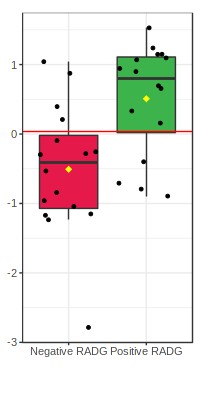

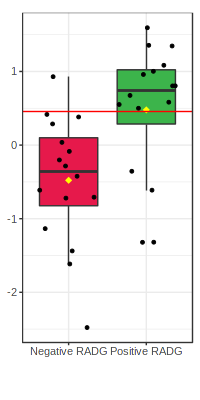

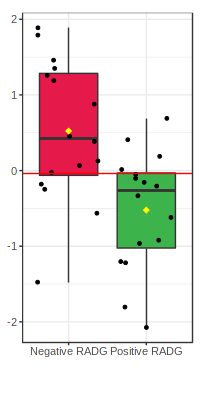


**Figure S3.** Relative distributions of the differentially abundant plasma metabolites in beef steers with divergent residual body weight gain phenotype.
